# Transcutaneous Electrical Acupoint Stimulation Combined with General Anesthesia in Minimally Invasive Surgery for Lung Cancer: A Randomized, Patient- and Assessor-Blinded, Sham-Controlled Trial

**DOI:** 10.3390/jcm15218627

**Published:** 2026-11-05

**Authors:** Yunxiao Zhang, Wanpu Yan, Jin Wei, Jing Wang, You Wan, Fengyu Liu, Jiheng Chen

**Affiliations:** 1 Key Laboratory of Carcinogenesis and Translational Research (Ministry of Education), Department of Anesthesiology, Peking University Cancer Hospital & Institute, Beijing 100142, China; yunxiao304@163.com (Y.Z.);; 2 Key Laboratory of Carcinogenesis and Translational Research (Ministry of Education), The First Department of Thoracic Surgery, Peking University Cancer Hospital & Institute, Beijing 100142, China; 3 Department of Neurobiology, School of Basic Medicine, Capital Medical University, Beijing 100069, China; 4 Key Laboratory for Neuroscience, Neuroscience Research Institute and Department of Neurobiology, School of Basic Medical Sciences, Ministry of Education/National Health Commission, Peking University, Beijing 100191, China

**Keywords:** general anesthesia, lung cancer, acupuncture, transcutaneous electrical acupoint stimulation, analgesia, electroencephalogram, neural network

## Abstract

**Background**: We aimed to clarify (1) the analgesic efficacy of transcutaneous electrical acupoint stimulation (TEAS) combined with general anesthesia during minimally invasive lung cancer surgery and (2) exploratory neural network correlates of acupuncture–drug combined anesthesia. **Methods**: Eligible patients receiving general anesthesia for minimally invasive lung cancer surgery were randomly allocated to perioperative TEAS or sham-TEAS in this randomized, patient- and assessor-blinded, sham-controlled trial. Postoperative patient-controlled intravenous analgesia (PCIA) was administered. Outcome measures included postoperative visual analog scale (VAS) pain scores, sufentanil consumption via PCIA, and total/effective PCIA pump presses. Perioperative electroencephalography (EEG) was acquired using a 22-lead recorder, and brainwave power spectra were analyzed using Fourier transform. Repeated-measures analysis of variance (RM-ANOVA) was applied to serial postoperative VAS scores at 6, 24 and 48 h. Independent-samples *t*-tests compared cumulative sufentanil consumption at 6, 24 and 48 h postoperatively. Post hoc simple-effect comparisons were performed as exploratory analyses without multiplicity adjustment. The EEG sub-study was exploratory; false-discovery-rate (FDR) correction was used to control multiple comparisons across electrodes and frequency bands. **Results**: In total, 109 patients were randomized. A significant group-by-time interaction was detected for postoperative VAS scores (F(1.599,171.124) = 68.287, *p* < 0.001). Simple-effect analyses revealed lower VAS scores in the TEAS group at postoperative 6 h (mean difference −1.900, 95%CI −2.181 to −1.619, *p* < 0.001) and 24 h (mean difference −1.391, 95%CI −1.645 to −1.138, *p* < 0.001), with no between-group difference at 48 h (mean difference −0.066, 95%CI −0.320 to 0.187, *p* = 0.605). The TEAS group also exhibited reduced PCIA sufentanil consumption at 6, 24 and 48 h postoperatively, alongside fewer total and effective PCIA pump presses. After FDR correction, exploratory EEG analysis identified potential dipole-related power differences in the prefrontal, occipital, parietal (postcentral gyrus), temporal and diencephalic regions, predominantly within beta-band oscillations (14–35 Hz). **Conclusions**: These exploratory findings suggest that TEAS combined with general anesthesia may reduce postoperative pain and analgesic requirement. This potential benefit may arise from TEAS-mediated modulation of pain-related neural networks; however, the observed intraoperative EEG oscillatory differences are exploratory neurophysiological correlates and do not confirm causal mechanisms.

## 1. Introduction

Acupuncture is widely used for sedative and analgesic purposes in traditional Chinese medicine [1]. In addition to conventional manual acupuncture, electrical acupuncture and transcutaneous electrical acupoint stimulation (TEAS) are used in clinical practice. TEAS combined with general anesthesia has been reported to reduce opioid consumption in cardiac surgery [2], to prevent postoperative nausea and vomiting (PONV) in high-risk patients undergoing laparoscopic gynecological surgery [3], and to reduce chronic pain after breast surgery [4]; our group previously reported TEAS analgesia after thoracoscopic lung cancer surgery [5]. However, the analgesic mechanism of this approach remains unclear.

Investigations into the mechanisms of action of acupuncture analgesia have focused on the peripheral mechanism and the lower central nervous system below the brain stem [6]. In recent years, it has become increasingly accepted that the generation and modulation of pain sensation also depends on the higher-level central nervous system, including the primary somatosensory cortex, secondary somatosensory cortex, insular cortex, anterior cingulate cortex, thalamus, and prefrontal cortex, among other regions of the brain, which form a neural network that modulates the pain sensation [7,8].

The primary mechanism of the neural network is neural oscillation. The combination of electroencephalogram (EEG) and neural signal calculation and analysis is effective for the study of neural oscillation. Our group previously demonstrated that patients with postherpetic neuropathic pain had increased gamma frequency (40–70 Hz) oscillation in the right parietal temporal region and that the power of gamma oscillation was positively correlated with the pain intensity of the patients. Furthermore, we showed that the gamma frequency band with increased power was located in the prefrontal cortex and cerebellum, regions related to pain perception, memory recall, and descending modulation [9]. Pain-related oscillatory signals can vary substantially across frequency bands and recording paradigms. That prior resting-state work focused on high-frequency gamma activity, which differs from the oscillatory signals explored in our intraoperative setting.

Although the existing literature suggests the brain modulates pain perception via distributed neural networks [7,8,9], whether and how acupuncture–drug compound anesthesia exerts its analgesic effects by acting on the neural network remain unclear. Our research group previously published a smaller-sample randomized controlled trial demonstrating that TEAS reduced postoperative pain and analgesic consumption after thoracoscopic lung cancer resection [5]. The present trial aimed to extend that prior work by recruiting a larger sample and adding an exploratory intraoperative EEG sub-analysis to probe potential neurophysiological correlates. We therefore sought to elucidate (1) the analgesic effect of transcutaneous electrical acupoint stimulation (TEAS) combined with general anesthesia in minimally invasive surgery for lung cancer and (2) exploratory neural network-related correlates of acupuncture–drug compound anesthesia.

**What is already known on this topic?** TEAS combined with general anesthesia can relieve postoperative pain and reduce postoperative analgesic drug consumption in patients undergoing thoracoscopic radical lung cancer surgery.

**What this study adds?** Building upon our previous smaller randomized controlled trial, the present exploratory trial provides further evidence consistent with a postoperative analgesic effect of TEAS with an enlarged sample size. In addition, we performed an exploratory EEG sub-analysis to identify potential neurophysiological correlates under general anesthesia. Observed power differences were mainly located in the prefrontal, occipital, parietal (postcentral gyrus), temporal lobe, and diencephalon brain regions, predominantly within the beta band (14–35 Hz). These findings should be interpreted as associative rather than causal mechanistic evidence.

**How this study might affect research, practice or policy?** This exploratory study reports an association between TEAS and postoperative analgesia; any role of pain-related neural network or emotional system brain regions remains hypothetical. Further fMRI and prospective studies are required to validate these exploratory neurophysiological observations.

## 2. Methods

### 2.1. Study Design

This is a single-center, prospective, randomized controlled trial, reported in accordance with the CONSORT 2010 statement; the completed CONSORT checklist is provided as supplementary material. The study protocol was approved by the Clinical Research Ethics Committee of Peking University Cancer Hospital in accordance with the Declaration of Helsinki (approval number: 2021kt44). Written informed consent was obtained from all participants prior to enrolment. This study was registered with the Chinese Clinical Trial Registry (http://www.chictr.org.cn; accession number: ChiCTR2100046940). Prospective registration date: 31 May 2021; first-patient enrolment: 1 July 2021.

The randomization sequence was generated by an independent statistician using computer-based random sampling. Allocation concealment was achieved using sequentially numbered opaque sealed envelopes. Patients, postoperative outcome assessors, EEG analysts, and statisticians were kept blinded to group allocation (patient- and assessor-blinded design). The operating surgeons and attending anesthesiologists in charge of intraoperative management were not blinded, given the nature of TEAS hardware setup. No formal post-intervention blinding-success questionnaire or perception assessment was performed. Prior to surgery, patients were informed that they would receive cutaneous electrical stimulation at acupoints, but they were not told whether they would receive high-intensity therapeutic stimulation or low-intensity sham stimulation. Participants could theoretically infer group assignment from the perceived stimulation intensity before anesthesia induction, creating a risk of partial unblinding and associated performance and detection bias. Our earlier trial [5] used a zero-stimulation sham condition, whereas in the present study, the sham-TEAS condition applied low-intensity 4 mA stimulation that only produced a faint cutaneous sensation; this intensity was selected because it was expected to produce minimal cutaneous sensation without established acupuncture-related analgesic effects, serving as a sham-control.

### 2.2. Participants

Patients were scheduled to undergo elective thoracoscopic radical resection for lung cancer in the First Department of Thoracic Surgery, Peking University Cancer Hospital; the recruitment period was from 1 July 2021 to January 2022. Inclusion criteria were age 18–64 years and being scheduled to undergo a lung operation for the first time. Exclusion criteria included unwillingness to receive acupoint electrical stimulation or EEG monitoring, skin infection at acupoints, upper or lower limb nerve injury, and inability to complete the research plan, including language difficulties, infectious diseases, and disorders of consciousness. Patients with a history of severe liver, kidney, brain, or lung disease, chronic pain, or alcohol or drug abuse were also excluded.

**Figure 1:**
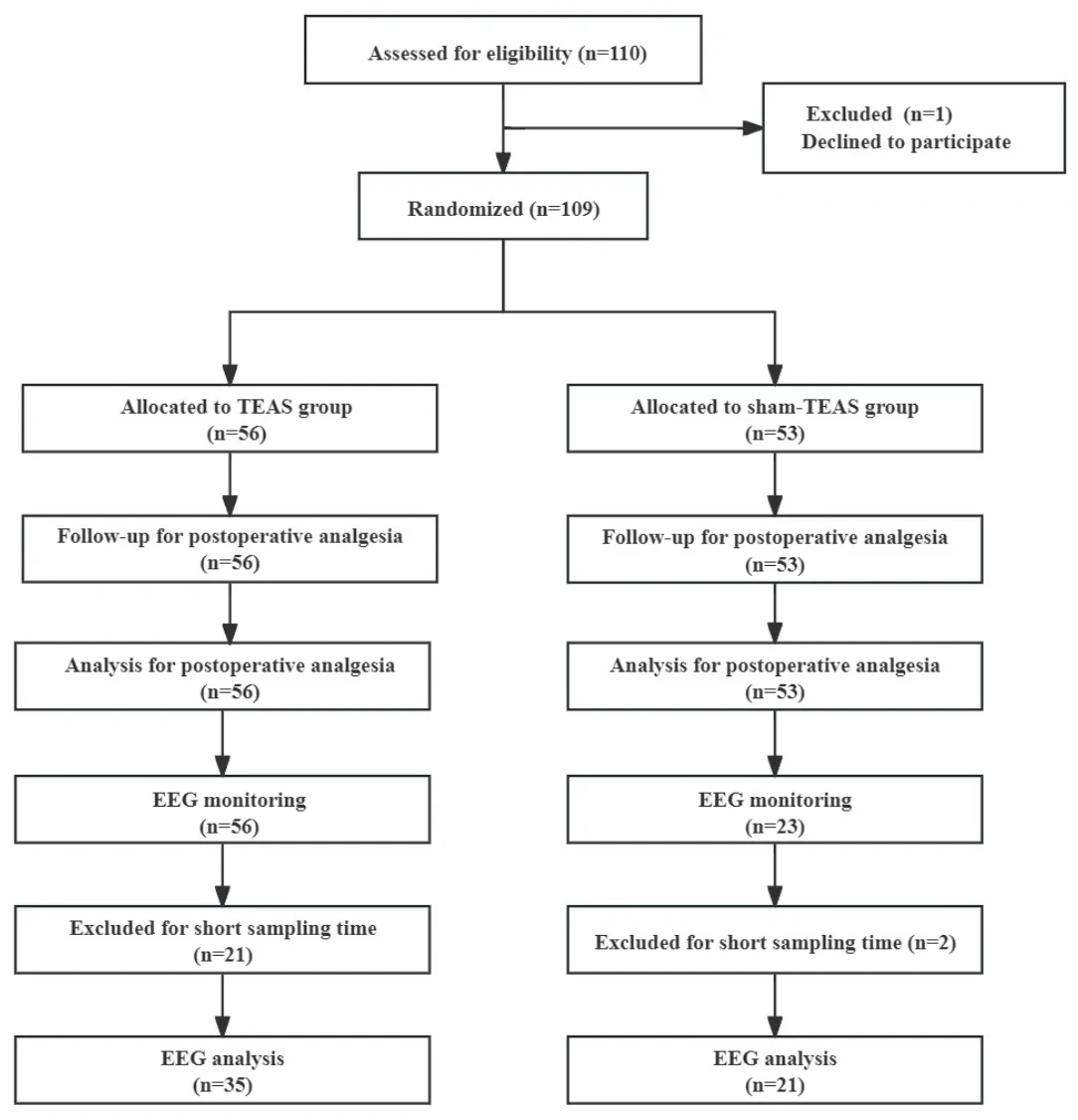
Study flowchart.

All screening exclusions took place prior to randomization. Only one participant withdrew consent after screening and before random assignment (shown in Figure 1).

### 2.3. TEAS Protocol

Enrolled patients were randomly assigned to receive either percutaneous electrical acupoint stimulation combined with general anesthesia (TEAS group) or percutaneous sham electrical acupoint stimulation plus general anesthesia (sham-TEAS group) in a 1:1 ratio by means of computer-based sample randomization. All patients were informed of the methods of TEAS and were anesthesia by a designated anesthesiologist 1 day before surgery. After arrival in the operating room, patients in both groups were connected to a HANS-200a transcutaneous acupoint nerve stimulator (Nanjing Jisheng Medical Technology, China) at the Hegu (LI4), Neiguan (PC6), Houxi (SI3) and Zhigou (TE6) acupoints on both sides. See Supplementary Figure S1 for an acupoint schematic diagram and Supplementary Figure S2 for the HANS-200a device photograph.

Patients in the TEAS group received TEAS for 30 min before induction of anesthesia, and TEAS was continued during the operation at a frequency of 2/100 Hz (2 Hz and 100 Hz alternating sparse-dense wave) and a stimulation intensity of 30 mA, producing a strong but tolerable cutaneous sensation. The stimulation frequency used in the sham-TEAS group was also 2/100 Hz, with a stimulation intensity of 4 mA, producing only a faint, minimal cutaneous sensation in conscious participants. The individual sensory threshold was not formally measured for each study participant before stimulation.

### 2.4. Anesthesia Protocol

All patients received total intravenous anesthesia. Sufentanil (0.3–0.5 µg/kg), propofol (2 mg/kg), and rocuronium (0.6–1 mg/kg) were used for induction of anesthesia. Mechanical ventilation (tidal volume, 6–8 mL/kg; respiratory rate, 12–15 times/min) was connected after endotracheal intubation. Propofol and remifentanil were infused continuously, and rocuronium was administered intermittently at a bi-spectral index of 40–60 and with hemodynamic parameters stable to maintain anesthesia. All operations were performed by the same surgeon, and the anesthesia management was also delivered by the same anesthesiologist following the standardized study-specific anesthesia protocol. For analgesia transition, 10 µg of sufentanil was administered 30 min before the end of the operation. After the operation, a patient-controlled intravenous analgesia (PCIA) pump was connected (sufentanil 1.5 μg/mL; priming volume, 0; PCIA dose, 2 mL (3 ug sufentanil); background infusion 1 mL/h; interval, 15 min; duration, 2 days). The flowchart is shown in Figure 1.

### 2.5. EEG Monitoring and Analysis

#### 2.5.1. EEG Data Acquisition

EEG recording was attempted for all 56 randomized TEAS participants. Due to limited intraoperative hardware and research-staff resources during clinical surgical workflow, it was not feasible to record EEG for every sham-TEAS subject. Twenty-three out of fifty-three sham-TEAS participants were selected using computer-generated simple random sampling without replacement for intraoperative EEG recording. This EEG sub-study was exploratory, and no pre-planned sample-size calculation was performed. All EEG analysts remained fully blinded to group allocation and clinical outcome data during data processing and artifact rejection. Recordings shorter than 180 s were excluded; exclusion was performed while analysts were blinded to group label and clinical outcome. Short valid recording segments were mainly attributed to short surgical duration, frequent electrosurgical artifacts and unstable intraoperative EEG signals.

EEG information was collected using a 22-channel EEG monitor (ANT Neuro, Hengelo, Netherlands). The electrode positioning for the 22 channels was based on the ANT WG standard 346 electrode position. Reference electrodes were placed on both ears, with impedance maintained below 5 KΩ; the sampling rate was 512 Hz. EEG was recorded intraoperatively under general anesthesia, and valid epochs were extracted from the anesthetic period after induction. All valid EEG epochs spanning the complete intraoperative anesthetic period from induction until skin closure were included; analyses were not restricted to predefined discrete surgical phases. Potential confounders, including anesthetic depth (BIS values), propofol and remifentanil infusion dosages, and electrosurgical artifacts, were managed in pre-processing steps: segments heavily contaminated by electrosurgery were manually marked and discarded prior to further decomposition.

#### 2.5.2. EEG Mapping Analysis

ADJUST is a fully automated algorithm for identifying and removing artifacts in EEG data; it is based on independent component analysis [10]. Independent component analysis was performed via the ADJUST plugin within EEGLAB. Default built-in threshold parameters of ADJUST were applied for artifact detection. The number of extracted independent components matched the number of EEG recording channels. ICs were accepted or rejected purely according to automatic ADJUST artifact-detection outputs without manual subjective adjustment. The EEG lead data were transformed into power values using Fourier analysis, and the EEG data were then decomposed into multiple independent components. On the basis of a series of characteristic parameters, independent components containing artifacts, such as blinking, eye movements, and discontinuous events, were identified and removed. The ADJUST plug-in can automate this process without a requirement for manual intervention or subjective judgment, allowing for the simple removal of artifacts from the data while leaving the components of neural source activity nearly unaffected. Afterward, a brain electrical activity map was generated on the basis of the location of the electrodes on the scalp.

#### 2.5.3. Dipole Analysis and Source Localization

Using the DIPFIT plug-in in EEGLAB (Swartz Center for Computational Neuroscience, La Jolla, CA, USA), a dipole was established for the components of independent component analysis to localize the source of the EEG signals and to further explore the specific brain regions and the direction of regulation of pain by means of electroacupuncture stimulation. Source localization was performed with DIPFIT plug-in. Three-shell boundary-element head model was adopted. Dipole acceptance criterion: residual variance <15%; dipoles with residual variance ≥15% were discarded from further analysis.

#### 2.5.4. Clustering Analysis

The obtained frequency information, dipole position information, and EEG topographic map information were subjected to principal component analysis, the data were reduced to three dimensions, the factors required for clustering were constructed with the same weight, and the clustering results of each principal component and dipole source localization were obtained by k-means clustering. The optimal k-means cluster number was determined by the elbow-criterion on PCA-reduced feature space. All independent components passing DIPFIT quality-control criteria entered into the k-means clustering workflow.

False-discovery-rate (FDR) correction was applied for multiple comparisons across electrodes and frequency bands. No further FDR correction was performed at the dipole-cluster level, either within individual clusters or across the full combined dataset of electrodes, frequency bands, and dipole clusters. Residual risk of type-I-error inflation remains for the high-dimensional EEG dataset.

### 2.6. Endpoints

The prespecified primary endpoints were the postoperative visual analog scale (VAS) pain score, and sufentanil consumption. The group-by-time interaction from RM-ANOVA represents a post hoc analytical approach not prespecified in the original statistical analysis plan. Secondary endpoints included postoperative total and effective PCIA pump attempts. Intraoperative EEG-related biomarkers represented post hoc exploratory secondary sub-study outcomes.

### 2.7. Sample Size

This exploratory randomized trial was prospectively registered in the Chinese Clinical Trial Registry (ChiCTR2100046940). According to the original trial protocol (28 April 2021), the prespecified primary outcome was postoperative VAS pain score, without a predefined single postoperative time-point. Sample-size calculation was driven by postoperative 6 h VAS pain score using effect-size estimates from our prior published work solely for power estimation; 6 h VAS was not designated as prespecified primary endpoint in registry documents. In previous studies, the VAS score at 6 h postoperatively was 3.1 ± 1.0 in the TEAS group and 5.7 ± 2.1 for the sham-TEAS group. At α = 0.05 two-sided, power = 0.8, the repeated-measures ANOVA module of PASS 11 was mistakenly selected instead of the independent-samples *t*-test module appropriate for a single time-point 6 h VAS comparison. Assumptions: 1:1 group allocation; anticipated common between-group standard deviations approximated from the prior study; anticipated 20% overall participant dropout rate. The calculated minimal required sample size was 45 participants per group; this 20% dropout allowance was applied once to the minimal per-group figure, yielding a planned target of 55 patients per group (total n = 110). Sample-size estimation was performed using PASS 11 software (NCSS, Kaysville, UT, USA). We note that this sample-size calculation was powered solely for the between-group comparison of 6 h postoperative VAS and was not powered for the post hoc RM-ANOVA group-by-time interaction analysis. No pre-planned sample-size calculation was performed for the exploratory EEG sub-study.

### 2.8. Statistical Analysis

Statistical analyses were performed using IBM SPSS Statistics (version 26.0; IBM Corp., Armonk, NY, USA). For postoperative visual analog scale (VAS) pain scores measured at postoperative 6 h, 24 h, and 48 h, repeated-measures analysis of variance (RM-ANOVA) was performed. Group allocation (TEAS versus sham-TEAS) was set as the between-subject factor and postoperative time-point served as the within-subject factor. Mauchly’s test of sphericity was applied to assess the sphericity assumption; Greenhouse–Geisser correction was adopted when the assumption was violated. Following a significant group-by-time interaction, post hoc simple-effect comparisons were conducted to evaluate inter-group differences at each postoperative time-point. Cumulative sufentanil consumption at postoperative 6 h, 24 h and 48 h was compared using independent-samples *t*-test. Given the nested mathematical relationship among these cumulative measures and the post hoc, unadjusted nature of these comparisons, all sufentanil consumption endpoints are treated as exploratory, and definitive clinical conclusions are not drawn from any single cumulative time-point. Categorical variables were analyzed using χ^2^ test or Fisher’s exact test as appropriate. For the exploratory EEG observational dataset, false-discovery-rate (FDR) correction was applied across electrodes and frequency bands. No FDR correction was performed within or across dipole-derived clusters, leaving a residual risk of type-I error inflation for the entire EEG dataset. A two-sided *p*-value < 0.05 was considered statistically significant.

## 3. Results

Patient characteristics are presented in Table 1, and no significant differences were found between the two groups in the baseline characteristics.

Repeated-measures analysis of variance (RM-ANOVA) was performed for postoperative VAS scores (6 h, 24 h, 48 h). Group (TEAS vs. sham-TEAS) was the between-subject factor and postoperative time was the within-subject factor.

Mauchly’s test revealed violation of sphericity assumption (W = 0.749, *p* < 0.001), so Greenhouse–Geisser correction (ε = 0.800) was applied. There were significant main effects of time (F_(1.599,171.124)_ = 513.202, *p* < 0.001) and group (F_(1,107)_ = 142.047, *p* < 0.001). A significant group-by-time interaction was observed (F_(1.599,171.124)_ = 68.287, *p* < 0.001), indicating distinct postoperative pain trajectories between groups.

**Figure 2:**
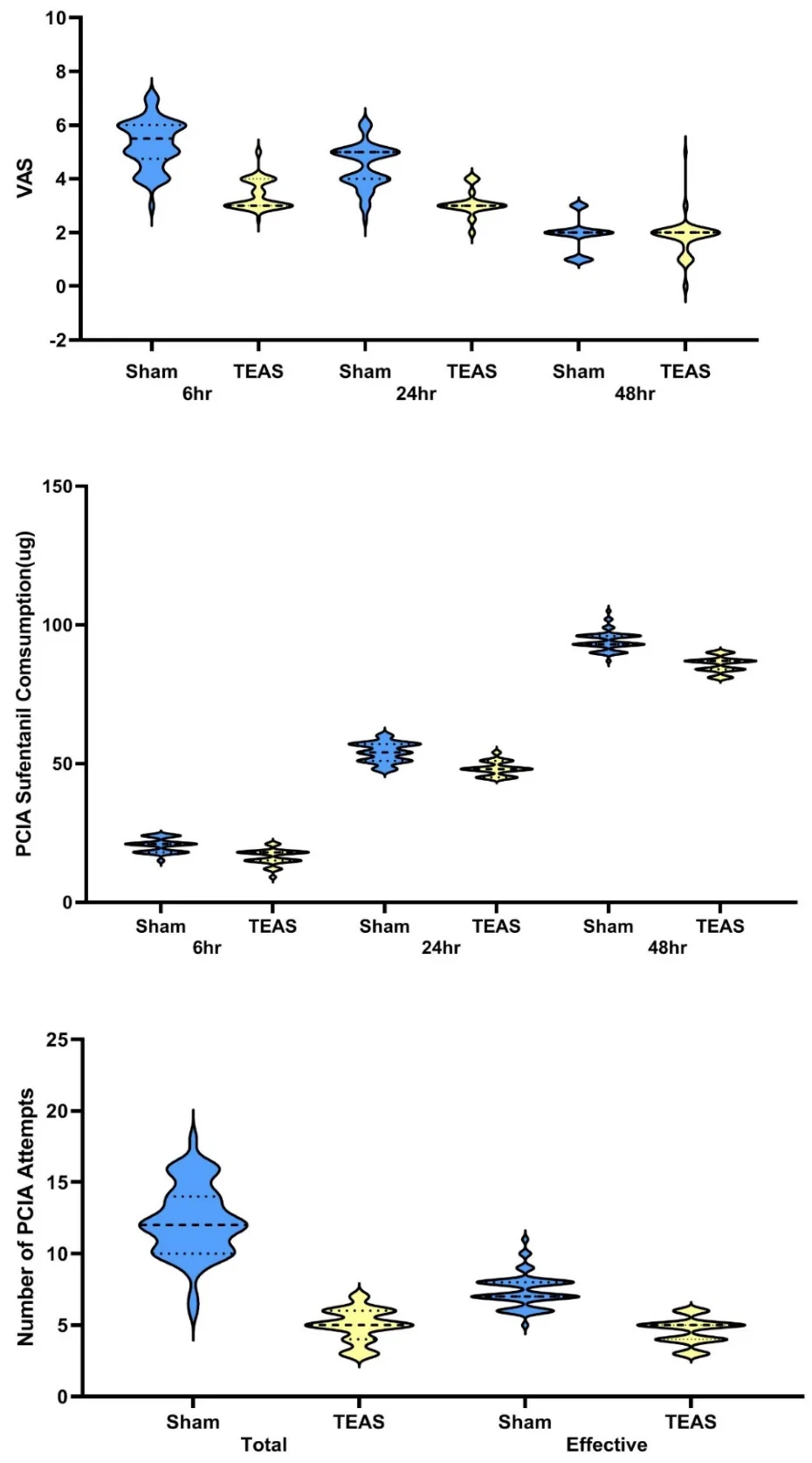
Postoperative pain scores, sufentanil consumption and PCIA pump attempts between TEAS and sham-TEAS groups. Postoperative visual analog scale (VAS) pain scores at 6 h, 24 h and 48 h after surgery. Data are presented as mean ± SD. Repeated-measures analysis of variance (RM-ANOVA) was used to assess group-by-time interaction. Cumulative postoperative sufentanil consumption at 6 h, 24 h and 48 h. Data are presented as mean ± SD, compared using independent-samples *t*-tests. Total and effective PCIA pump attempts. Data are presented as mean ± SD, compared using independent-samples *t*-tests. TEAS, transcutaneous electrical acupoint stimulation; PCIA, patient-controlled intravenous analgesia; VAS, visual analog scale.

Subsequent simple-effect analyses showed significant inter-group differences at postoperative 6 h and 24 h, but no difference at 48 h. At 6 h, mean VAS was 3.4 ± 0.6 for TEAS versus 5.3 ± 0.9 for sham-TEAS (mean difference = −1.900, 95%CI −2.181 to −1.619, *p* < 0.001). At 24 h, VAS was 3.1 ± 0.5 versus 4.5 ± 0.8 (mean difference = −1.391, 95%CI −1.645 to −1.138, *p* < 0.001). At 48 h, VAS was 1.8 ± 0.7 versus 1.9 ± 0.6 (mean difference = −0.066, 95%CI −0.320 to 0.187, *p* = 0.605). Changes in VAS score are shown in Figure 2, and the VAS trajectories are presented in Supplementary Figure S3.

Cumulative sufentanil consumption at postoperative 6 h, 24 h and 48 h was compared using an independent-samples *t*-test. The consumption of sufentanil during PCIA was significantly lower in the TEAS group than in the sham-TEAS group at 6 h (16.3 ± 2.8 vs. 20.5 ± 2.5 μg; *p* < 0.001), 24 h (48.2 ± 2.5 vs. 54.0 ± 3.5 μg; *p* < 0.001), and 48 h (85.8 ± 2.8 vs. 94.2 ± 3.4 μg; *p* < 0.001) after surgery (Figure 2). The mean number of total PCIA pump attempts was significantly lower in the TEAS group than in the sham-TEAS group (5.0 ± 1.1 vs. 12.3 ± 2.5; *p* < 0.001), as were the mean effective attempts (4.6 ± 0.9 vs. 7.4 ± 1.1; *p* < 0.001) (Figure 2).

Postoperative adverse-event outcomes were also systematically collected. No significant differences were seen in postoperative nausea and vomiting, respiratory depression, dizziness, hypotension and local skin irritation between the two groups (Supplementary Table S1).

### 3.1. EEG Power Spectrum Results

#### Data Collection

Data were collected from 79 patients (56 in the TEAS group and 23 in the sham-TEAS group). Data with a short sampling time (<180 s) were removed. Finally, data on 56 patients were retained: 35 in the TEAS group and 21 in the sham-TEAS group. Baseline comparisons between the EEG sub-cohort versus full randomized cohort, and comparisons between usable-EEG and excluded-EEG participants showed no significant differences (Supplementary Tables S2 and S3). The retained EEG data were separately filtered using finite impulse response filters for 1–40 Hz bandpass filtering, and data segments with amplitudes >100 µV were deleted. The average reference was reset, and other pre-processing was performed. Power spectral analysis was performed across six predefined frequency bands within the 1–40 Hz passband: delta (1.0–3.5 Hz), theta (4.0–7.5 Hz), alpha-1 (8.0–10.5 Hz), alpha-2 (11.0–13.0 Hz), beta-1 (14.0–24.0 Hz), and beta-2 (25.0–35.0 Hz). Frequencies above 35 Hz were visualized, but were not subjected to between-group statistical comparisons.

### 3.2. Power Spectral Results

**Figure 3:**
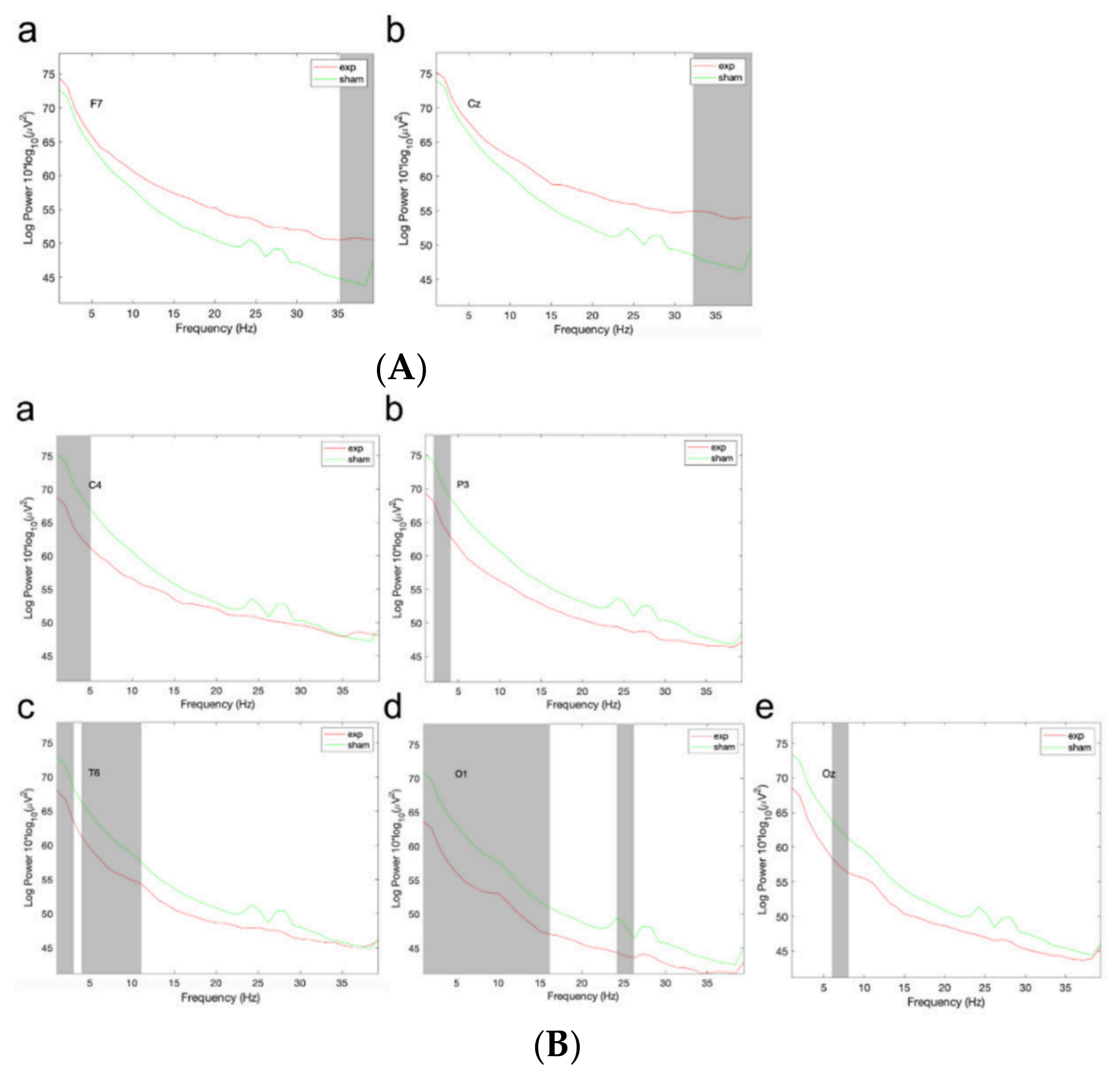
Power spectral density diagram of F7 (**a**) and Cz (**b**) channels (**A**). Power density diagram of C4 (**a**), P3 (**b**), T6 (**c**), O1 (**d**), and Oz (**e**) channels (**B**). Spectra are plotted over the 1–40 Hz filter passband. Statistical comparisons were limited to predefined frequency bands ≤ 35 Hz.

The spectral maps of the TEAS group and the sham-TEAS group were calculated separately and then compared. Statistically significant differences were observed for some frequency ranges of the F7, Cz, C4, P3, T6, O1, and Oz channels. For instance, in the high-frequency part of the F7 and Cz channels, the TEAS group had significantly higher power (Figure 3A). In the low-frequency part of the C4, P3, T6, O1, and Oz channels, the TEAS group had significantly lower power (Figure 3B).

### 3.3. Frequency Distribution Statistics of Each Cluster

**Figure 4:**
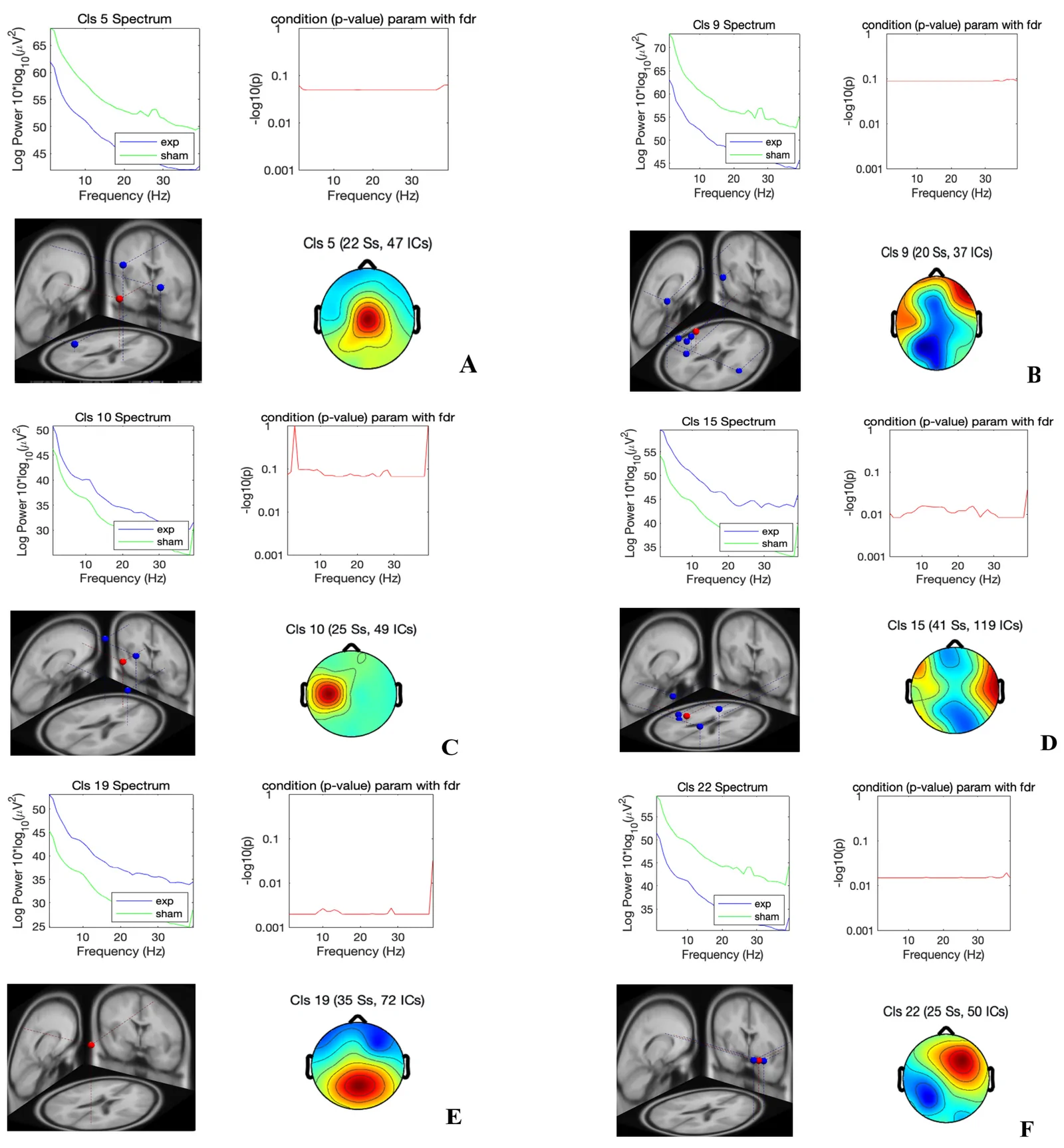
Exploratory EEG power differences between TEAS and sham-TEAS groups. Panels (**A**–**F**) each contain four sub-panels: power-spectrum plot (**top-left**), −log_10_(p) curve with FDR significance threshold line (**top-right**), dipole source localization map (**bottom-left**), and scalp topographic map (**bottom-right**). In power-spectrum plots, the y-axis represents log-transformed EEG oscillatory power (10*log_10_(μV^2^)) and the x-axis represents frequency (Hz). In the −log_10_(p) plots, the horizontal red line denotes the FDR-adjusted significance threshold; points above this red line indicate statistically significant group differences. Each panel label includes cluster identifier (Cls), the number of included subjects (Ss), and the number of independent components (ICs) for that dipole-derived cluster. EEG power spectra were calculated using Fourier transform. False-discovery-rate (FDR) correction was applied across electrodes and frequency bands for multiple comparison control. No FDR correction was performed within or across dipole-derived clusters, leaving residual risk of type-I error inflation. Regions showing between-group differences were predominantly observed within beta-band oscillations (14–35 Hz). TEAS, transcutaneous electrical acupoint stimulation; EEG, electroencephalogram; Ss, subjects; ICs, independent components.

The power of dipoles located in the prefrontal, occipital, and parietal lobes (posterior central gyrus) was higher and the power of dipole electric activity in the temporal lobe and diencephalon was lower in the TEAS group than in the control group; oscillations were primarily observed in the beta band (Figure 4A–F). These are exploratory associative findings from an unbalanced post hoc EEG sub-study and cannot constitute causal mechanistic proof of analgesic pathways. The frequency information for each cluster, EEG topographic map information, and dipole position information are shown in Supplementary Figures S1–S3.

## 4. Discussion

In this randomized, patient- and assessor-blinded, sham-controlled trial, we found that perioperative TEAS reduced postoperative pain intensity and opioid consumption after minimally invasive lung cancer surgery, which was in accordance with our previous small-sample-size study [5].

The electroacupuncture stimulation technique used in this study is currently the most commonly used TEAS technique in clinical practice. Since previous research has shown that sparse-dense wave stimulation can promote the release of three endogenous opioids (peptides endorphin, enkephalin, and dynorphin) and delay the development of analgesia tolerance under continuous TEAS treatment [11,12,13,14], we used 2 Hz and 100 Hz alternating sparse-dense waves. Owing to its extensive and long-lasting analgesic effect caused by the activation of fine Aδ and C fibers and subsequent negative feedback modulation in the brain stem, strong electroacupuncture stimulation is more effective than weak electroacupuncture stimulation. A recent TEAS study protocol in older urologic laparoscopic surgery set active stimulation at 2–3 times each participant’s sensory threshold (capped at 15 mA) and used a fixed 4 mA sensory-level sham [15]; we adopted an analogous 4 mA fixed sham intensity in the present trial. We did not, however, titrate the active intensity to individual sensory thresholds, applying a fixed 30 mA instead. In accordance with the meridian theory of traditional Chinese medicine, Hegu (LI4), Neiguan (PC6), and Houxi (SI3) can be used for analgesia in various parts of the body, whereas Zhigou (TE6) is mainly used for relief of flank and rib pain. The combination of the above four acupoints can balance the functions of the internal organs by regulating the mind and qi, and therefore Hegu (LI4), Neiguan (PC6), Houxi (SI3), and Zhigou (TE6) were selected as the acupoints for the present study.

We also investigated exploratory neurophysiological correlates of acupuncture–drug compound anesthesia by use of EEG monitoring and analysis. Between-group differences in specific frequency ranges were observed; these should be regarded as exploratory associations of EEG activity recorded under general anesthesia during acupoint stimulation, not as evidence that TEAS changes cortical activity. With the exception of eye electricity and electromyography clusters, the total power spectra with and without electroacupuncture stimulation differed among clusters. The power of dipole activity in the prefrontal, occipital, and parietal lobes (posterior central gyrus) was higher and the power in the temporal lobe and diencephalon was lower in the TEAS group, with group differences mainly occurring in the beta band. We emphasize that EEG was recorded under deep general anesthesia, and brain oscillations are heavily confounded by propofol, remifentanil dose, anesthetic depth, surgical manipulation, electrosurgical artifacts, and physiological fluctuation; hence, source-localization outputs should be interpreted cautiously as associative observations rather than mechanistic causal evidence. We cannot conclude that TEAS “enhances the ability of the brain to perceive and process pain signals and therefore achieves pain relief”; such causal inference cannot be supported by this EEG sub-analysis. We only report observed between-group oscillatory associations.

Human pain processing is widely described as a multi-component system, encompassing sensory-discriminative signal transduction, affective-motivational, and cognitive-evaluative dimensions [16]. Our observed pattern of oscillatory power alterations across multiple cortical and subcortical regions partially overlaps with the known brain regions participating in human pain processing, including somatosensory, prefrontal, temporal, and diencephalic structures. Nevertheless, given the exploratory nature of this EEG sub-study, we cannot draw firm mechanistic conclusions regarding how TEAS modulates these distributed brain oscillations. The observed beta band power differences should be regarded as correlative neurophysiological observations rather than proof of specific pain-processing pathways. Our findings also reveal that between-group differences in EEG activity were mainly focused on the beta band (14–35 Hz). Electroacupuncture stimulation may cause changes in oscillations in the beta band in the brain by stimulating specific acupoints on the skin, thereby regulating the release of neurotransmitters, neuropeptides, and other substances and affecting the brain’s transmission and processing of pain signals. This remains a hypothetical interpretation.

This study has several limitations. First, this was a single-center randomized controlled study with a relatively modest sample size. Participants were enrolled within a relatively narrow age range, which may restrict the generalizability of our findings to other patient populations or diverse surgical contexts. Second, postoperative VAS pain scores were assessed at discrete time-points, and post hoc simple-effect comparisons at individual time-points were exploratory and not adjusted for multiple testing, which may inflate the risk of type I error; the RM-ANOVA group-by-time interaction itself was a post hoc analysis and was not the prespecified primary inference. Likewise, comparisons of 6 h and 24 h cumulative sufentanil consumption are exploratory without multiplicity adjustment, and the 48 h cumulative sufentanil endpoint was likewise designated as post hoc; definitive clinical conclusions should not rest on this post hoc endpoint alone. Third, blinding success was not formally assessed. We cannot fully exclude the possibility that participants might guess group assignment based on perceived stimulation intensity before anesthesia induction. Moreover, the 4 mA sham-TEAS stimulus may exert weak biological effects and cannot be guaranteed to be entirely biologically inert. Fourth, the EEG analysis was an exploratory secondary sub-study. EEG recording was not obtained for all randomized participants, and substantial numbers of EEG recordings were excluded due to insufficient recording duration, resulting in small and imbalanced sample sizes between the TEAS and sham-TEAS groups. This post-randomization subset carries potential selection bias for mechanistic interpretation. No predefined sample-size calculation was performed for the EEG sub-study. We applied FDR correction for multiple comparisons across electrodes and frequency bands, but no additional FDR correction was implemented at the dipole-cluster level, leaving residual type-I error inflation risk for this high-dimensional neurophysiological dataset. In addition, source-localization outputs from intraoperative EEG have inherently limited reproducibility. Fifth, we cannot fully exclude confounding influences from anesthetic depth, intraoperative medications, and surgical stimulation on perioperative EEG signals. This analysis cannot disentangle direct TEAS-related neural modulation from indirect effects mediated by reduced surgical stress or opioid-sparing effects. Finally, this study focuses exclusively on short-term postoperative pain-related endpoints. We lack the long-term follow-up data to evaluate whether TEAS produces sustained effects on pain-related neural networks or persistent clinical benefits.

In conclusion, findings from this exploratory randomized trial suggest that TEAS combined with general anesthesia may produce lower postoperative pain and reduced postoperative analgesic consumption. Any involvement of pain-related neural networks is not established; the intraoperative EEG oscillatory differences reported here represent exploratory neurophysiological correlates rather than causal mechanistic proof.

## Figures and Tables

**Table 1:** Characteristics of the study patients (n = 109).

Characteristic	TEAS (n = 56)	Sham-TEAS (n = 53)	*p* Value
Age (years)	57.1 ± 4.6	57.7 ± 4.6	0.481
Sex, n (%)			0.791
Female	31 (55.4)	28 (52.8)	
Male	25 (44.6)	25 (47.2)	
BMI (kg/m^2^)	21.8 ± 0.9	22.0 ± 1.4	0.387
Procedure, n (%)			0.435
Single	48 (85.7)	48 (90.6)	
Bilobectomy	8 (14.3)	5 (9.4)	
Duration of surgery (min)	105.9 ± 16.9	111.1 ± 13.4	0.066
Dose of propofol (mg)	963.9 ± 59.8	954.2 ± 60.5	0.398
Dose of remifentanil (mg)	1.3 ± 0.2	1.2 ± 0.2	0.368
Dose of rocuronium bromide (mg)	96.9 ± 20.3	99.6 ± 16.0	0.436
Histological subtype, n (%)			0.454
Adenocarcinoma	43 (76.8)	44 (83.0)	
Squamous-cell carcinoma	12 (21.4)	7 (13.2)	
Others ^a^	1 (1.8)	2 (3.8)	
Tumor maximum diameter (cm), mean ± SD	3.1 ± 0.5	3.2 ± 0.5	0.330
Lymph-node-dissection pattern, n (%)			0.676
Systematic mediastinal lymph-node dissection	37 (66.1)	37 (69.8)	
Lymph-node sampling	19 (33.9)	16 (30.2)	

Data are expressed as number (%) or mean ± standard deviation. BMI, body mass index; TEAS, transcutaneous electrical acupoint stimulation. ^a^ includes adenosquamous carcinoma and large-cell carcinoma.

## Data Availability

The trial protocol, CONSORT checklist, and statistical analysis plan are available as supplementary materials. De-identified study data can be obtained from the corresponding author upon reasonable written request, subject to institutional ethics-board approval. Raw EEG data are not publicly shared due to restrictions imposed by local ethics regulations.

## Supplementary Materials

The following supporting information can be downloaded at https://www.mdpi.com/article/10.3390/jcm15218627/s1. Figure S1: Schematic diagram of acupoint locations for transcutaneous electrical acupoint stimulation (TEAS); Figure S2: The HANS‑200A transcutaneous electrical acupoint stimulation (TEAS) device; Supplementary Figure S3: The VAS trajectories between TEAS and Sham-TEAS; Table S1: Postoperative adverse‑event outcomes; Table S2: Baseline comparison between participants with EEG recordings and those without EEG recordings within the full randomized cohort; Table S3: Baseline comparison between participants with usable EEG data and those excluded from EEG analysis.

## References

[1] Wu M.-S., Chen K.-H., Chen I.-F., Huang S.-K., Tzeng P.-C., Yeh M.-L., Lee F.-P., Lin J.-G., Chen C. (2016). The efficacy of acupuncture in post-operative pain management: A systematic review and meta-analysis. PLoS ONE.

[2] Zhang H., Wang L., Zheng Z., Han J., Li L., Yao W., Li Z., Luo G., Gao B., Shen J. (2024). The use of transcutaneous electrical acupoint stimulation to reduce opioid consumption in patients undergoing off-pump CABG: A randomized controlled trial. Perioper. Med..

[3] Qin J., Ye X., Ye C., Huang X., Sun H., Zhao X., Tong Y., Mazomba M., Mo Y. (2023). The Effect of Transcutaneous Electrical Acupoint Stimulation on High-Risk Patients with PONV Undergoing Laparoscopic Gynecologic Surgery: A Randomized Controlled Trial. J. Clin. Med..

[4] Lu Z., Wang Q., Sun X., Zhang W., Min S., Zhang J., Zhao W., Jiang J., Wang Y., Zhu Y. (2021). Transcutaneous electrical acupoint stimulation before surgery reduces chronic pain after mastectomy: A randomized clinical trial. J. Clin. Anesth..

[5] Chen J., Zhang Y., Li X., Wan Y., Ji X., Wang W., Kang X., Yan W., Fan Z. (2020). Efficacy of transcutaneous electrical acupoint stimulation combined with general anesthesia for sedation and postoperative analgesia in minimally invasive lung cancer surgery: A randomized, double-blind, placebo-controlled trial. Thorac. Cancer.

[6] Lin J.-G., Kotha P., Chen Y.-H. (2022). Understandings of acupuncture application and mechanisms. Am. J. Transl. Res..

[7] Cai R.-L., Shen G.-M., Wang H., Guan Y.-Y. (2018). Brain functional connectivity network studies of acupuncture: A systematic review on resting-state fMRI. J. Integr. Med..

[8] Sun R., Yang Y., Li Z., Li Y., Cheng S., Zeng F. (2014). Connectomics: A new direction in research to understand the mechanism of acupuncture. Evid. Based Complement. Altern. Med..

[9] Zhou R., Wang J., Qi W., Liu F.-Y., Yi M., Guo H., Wan Y. (2018). Elevated resting state gamma oscillatory activities in electroencephalogram of patients with post-herpetic neuralgia. Front. Neurosci..

[10] Mognon A., Jovicich J., Bruzzone L., Buiatti M. (2011). ADJUST: An automatic EEG artifact detector based on the joint use of spatial and temporal features. Psychophysiology.

[11] Chen X.-H., Han J.-S. (1992). Analgesia induced by electroacupuncture of different frequencies is mediated by different types of opioid receptors: Another cross-tolerance study. Behav. Brain Res..

[12] DeSantana J.M., da Silva L.F.S., Sluka K.A. (2010). Cholecystokinin receptors mediate tolerance to the analgesic effect of TENS in arthritic rats. Pain.

[13] Chandran P., Sluka K.A. (2003). Development of opioid tolerance with repeated transcutaneous electrical nerve stimulation administration. Pain.

[14] Desantana J.M., Santana-Filho V.J., Sluka K.A. (2008). Modulation between high- and low-frequency transcutaneous electric nerve stimulation delays the development of analgesic tolerance in arthritic rats. Arch. Phys. Med. Rehabil..

[15] Liu X., Fan Y., Wu C., Hu R., Wang T., Cao Y., Liu G., Zhang L., Han T. (2025). Effect of Transcutaneous Electrical Acupoint Stimulation on Postoperative Sleep Disorders in Older Patients After Urologic Laparoscopic Surgery by Regulating Intestinal Microbiota: Study Protocol for a Prospective Controlled Trial. Nat. Sci. Sleep.

[16] Price D.D. (2000). Psychological and neural mechanisms of the affective dimension of pain. Science.

