# Supplementary material for: Transcutaneous Electrical Acupoint Stimulation Combined with General Anesthesia in Minimally Invasive Surgery for Lung Cancer: A Randomized, Patient- and Assessor-Blinded, Sham-Controlled Trial
Source: J Clin Med. 2026 Nov 5;15(21):8627. doi: 10.3390/jcm15218627 (PMC13764418; doi:10.3390/jcm15218627)
Supplement: Supplementary file 1 [file jcm-15-08627-s001.zip › Supplementary Figure S1.pdf]

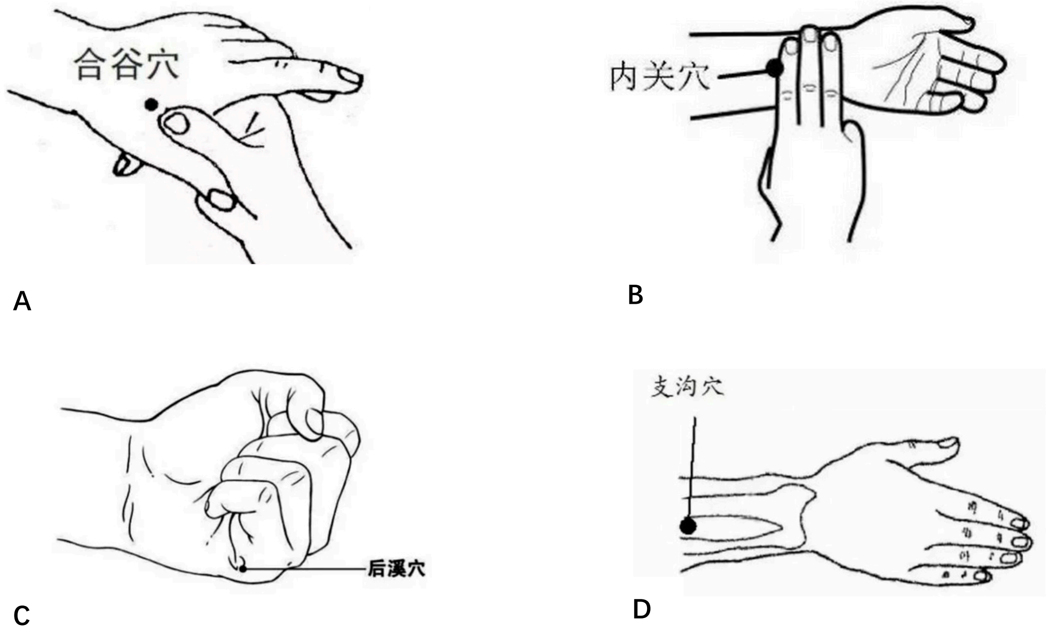

Supplementary Figure S1 Schematic diagram of acupoint locations for transcutaneous electrical acupoint stimulation (TEAS) (A) Hegu (LI4); (B) Neiguan (PC6); (C) Houxi (SI3); (D) Zhigou (TE6). Black solid dots mark acupoints defined according to Chinese national standard GB/T 12346-2021. (A) Hegu (LI4) is located on the radial midpoint of the 2nd metacarpal bone between the 1st and 2nd metacarpals. (B) Neiguan (PC6) is located 2 cun proximal to the distal palmar wrist crease, between the palmaris longus tendon and flexor carpi radialis tendon. (C) Houxi (SI3) is located on the ulnar side of the palm with a slightly clenched fist, in the depression at the end of the distal palmar crease posterior to the 5th metacarpophalangeal joint. (D) Zhigou (TE6) is located 3 cun proximal to the dorsal wrist crease, in the gap between the ulna and radius.
