# Supplementary material for: Transcutaneous Electrical Acupoint Stimulation Combined with General Anesthesia in Minimally Invasive Surgery for Lung Cancer: A Randomized, Patient- and Assessor-Blinded, Sham-Controlled Trial
Source: J Clin Med. 2026 Nov 5;15(21):8627. doi: 10.3390/jcm15218627 (PMC13764418; doi:10.3390/jcm15218627)
Supplement: Supplementary file 1 [file jcm-15-08627-s001.zip › Supplementary Figure S2.pdf]

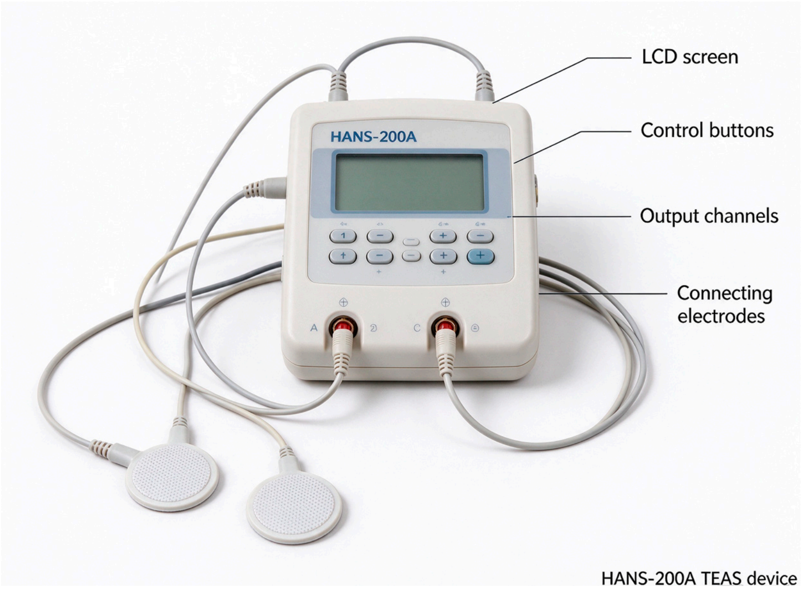


**Supplementary Figure S2 The HANS-200A transcutaneous electrical acupoint stimulation (TEAS) device.** The HANS-200A TEAS apparatus (Nanjing Jisheng Medical Technology Co., Ltd., Nanjing, China) applied in this randomized trial.
