# Supplementary material for: Transcutaneous Electrical Acupoint Stimulation Combined with General Anesthesia in Minimally Invasive Surgery for Lung Cancer: A Randomized, Patient- and Assessor-Blinded, Sham-Controlled Trial
Source: J Clin Med. 2026 Nov 5;15(21):8627. doi: 10.3390/jcm15218627 (PMC13764418; doi:10.3390/jcm15218627)
Supplement: Supplementary file 1 [file jcm-15-08627-s001.zip › Supplenmentary Table S1.pdf]

Supplementary Table S1 Postoperative adverse-event outcomes.

Characteristic	TEAS (n=56)	Sham-TEAS (n=53)	P Value
postoperative nausea and vomiting (PONV) [a]	6	13	0.057
respiratory depression [b]	2	2	0.955
Dizziness [c]	3	3	0.945
Hypotension [d]	5	4	0.793
local skin irritation at TEAS electrode sites [e]	2	0	0.165

Data are expressed as number (%). TEAS, transcutaneous electrical acupoint stimulation.

[a] Subjective feeling of nausea or any episode of vomiting within 48 hours after surgery.

[b] Respiratory rate less than 10 breaths per minute or oxygen saturation < 93% on room air, requiring clinical intervention.

[c] Self-reported lightheadedness or vertigo occurring within 48 hours postoperatively.

[d] Systolic blood pressure < 90 mmHg or a ≥ 20% reduction from baseline systolic blood pressure, recorded within 48 hours after surgery.

[e] Erythema, pruritus, rash or mild swelling at the skin area where transcutaneous electrical acupoint stimulation electrodes were applied.
