# Supplementary material for: Transcutaneous Electrical Acupoint Stimulation Combined with General Anesthesia in Minimally Invasive Surgery for Lung Cancer: A Randomized, Patient- and Assessor-Blinded, Sham-Controlled Trial
Source: J Clin Med. 2026 Nov 5;15(21):8627. doi: 10.3390/jcm15218627 (PMC13764418; doi:10.3390/jcm15218627)
Supplement: Supplementary file 1 [file jcm-15-08627-s001.zip › Supplenmentary Table S2.pdf]

Supplementary Table S2 Baseline comparison between participants with EEG recordings and those without EEG recordings within the full randomized cohort.

**Characteristic**	**With EEG recordings (n=56)**	**Without EEG recordings (n=53)**	**P Value**
Age (years)	57.0 ± 4.7	57.7 ± 4.5	0.430
Sex, n (%)			0.614
Female	27	23	
Male	29	30	
BMI (kg/m^2)	21.9 ± 1.1	21.8 ± 1.3	0.571
Procedure			0.849
Single	49	47	
Bilobectomy	7	6	
Duration of surgery (min)	108.4 ± 16.5	108.8 ± 14.5	0.869
Dose of propofol (mg)	966.6 ± 67.7	951.4 ± 50.2	0.187
Dose of remifentanil (mg)	1.3 ± 0.3	1.2 ± 0.2	0.668
Histological subtype, n (%)			0.692
Adenocarcinoma	44	43	
Squamous-cell carcinoma	11	8	
Others [a]	1	2	
Tumour maximum diameter (cm), mean ± SD	3.2 ± 0.5	3.1 ± 0.5	0.220
Lymph-node-dissection pattern, n (%)			0.407
Systematic mediastinal lymph-node dissection	36	38	
Lymph-node sampling	20	15	

Data are expressed as number (%) or mean ± standard deviation. BMI, body mass index; TEAS, transcutaneous electrical acupoint stimulation.

[a] include adenosquamous carcinoma and large-cell carcinoma.
