# Supplementary material for: Transcutaneous Electrical Acupoint Stimulation Combined with General Anesthesia in Minimally Invasive Surgery for Lung Cancer: A Randomized, Patient- and Assessor-Blinded, Sham-Controlled Trial
Source: J Clin Med. 2026 Nov 5;15(21):8627. doi: 10.3390/jcm15218627 (PMC13764418; doi:10.3390/jcm15218627)
Supplement: Supplementary file 1 [file jcm-15-08627-s001.zip › Supplenmentary Table S3.pdf]

Supplementary Table S3 Baseline comparison between participants with usable EEG data and those excluded from EEG analysis.

Characteristic	With usable EEG data (n=56)	Excluded from EEG analysis (n=23)	P Value
Age (years)	57.0 ± 4.7	57.4 ± 4.8	0.790
Sex, n (%)			0.461
Female	27	9	
Male	29	14	
BMI (kg/m^2)	22.0 ± 1.1	22.1 ± 0.9	0.445
Procedure, n (%)			0.568
Single	49	19	
Bilobectomy	7	4	
Duration of surgery (min)	108.4 ± 16.5	103.8 ± 14.7	0.258
Dose of propofol (mg)	966.6 ± 67.7	952.5 ± 49.4	0.369
Dose of remifentanil (mg)	1.3 ± 0.3	1.2 ± 0.2	0.273
Histological subtype, n (%)			0.800
Adenocarcinoma	44	18	
Squamous-cell carcinoma	11	5	
Others [a]	1	0	
Tumour maximum diameter (cm), mean ± SD	3.2 ± 0.5	3.0 ± 0.6	0.142
Lymph-node-dissection pattern, n (%)			0.653
Systematic mediastinal lymph-node dissection	36	16	
Lymph-node sampling	20	7	

Data are expressed as number (%) or mean ± standard deviation. BMI, body mass index; TEAS, transcutaneous electrical acupoint stimulation.

[a] include adenosquamous carcinoma and large-cell carcinoma.
